# Septic arthritis of the knee from a peripheral venous catheter infection in an extremely low birthweight infant

**DOI:** 10.1111/ped.70237

**Published:** 2025-10-14

**Authors:** Ai Higuchi, Masashi Ota, Kazumi Morisawa, Munehiro Furuichi, Takeshi Arimitsu, Tatsuaki Matsumoto, Mariko Hida

**Affiliations:** ^1^ Department of Pediatrics Keio University School of Medicine Tokyo Japan; ^2^ Department of Orthopedic Surgery Keio University School of Medicine Tokyo Japan

**Keywords:** extremely low birthweight, neonatal intensive care unit, peripheral venous catheter, septic arthritis

Septic arthritis is rare in neonates but can lead to severe long‐term complications including joint dislocation and laxity, limited mobility, and limb‐length discrepancy.^1^ Early diagnosis and treatment are crucial, as delayed treatment increases the risk of complications.^1^, ^2^

Hematogenous spread is responsible for most septic arthritis in neonates. While central venous catheters (CVCs) and umbilical vascular catheters are established risk factors,^3^ infection from peripheral venous catheters (PVCs) is not widely recognized as a significant risk factor. We experienced a rare case of septic arthritis triggered by a skin infection at a PVC insertion site.

An 858‐g male neonate (−3.75 SD) was born at 32 weeks and 4 days of gestation via emergency cesarean section because of late‐onset transient bradycardia. He was suspected of fetal growth restriction due to maternal hypertensive disorders of pregnancy (HDP). Apgar scores were 6 and 9 at 1 and 5 min, respectively. No abnormalities were detected on physical examination or newborn screening.

A peripherally inserted central catheter (PICC) was placed in his right hand after birth and removed on the day of life 13. Enteral feeding progressed, but on the day of life 18, abdominal distension was observed. PVC was placed in the dorsum of his left hand from the day of life 18 to 21. Catheter management in our neonatal intensive care unit (NICU) followed the Guidelines for the Prevention of Intravascular Catheter‐Related Infections,^4^ including sterile insertion and regular site monitoring. On the day of life 23, 2 days after the removal of PVC, redness and swelling accompanied by an accumulation of pus were found at the PVC site (Figure 1a). Daily drainage and bacitracin application improved the lesion. Methicillin‐susceptible *Staphylococcus aureus* (MSSA) was isolated from the pus.

**FIGURE 1:**
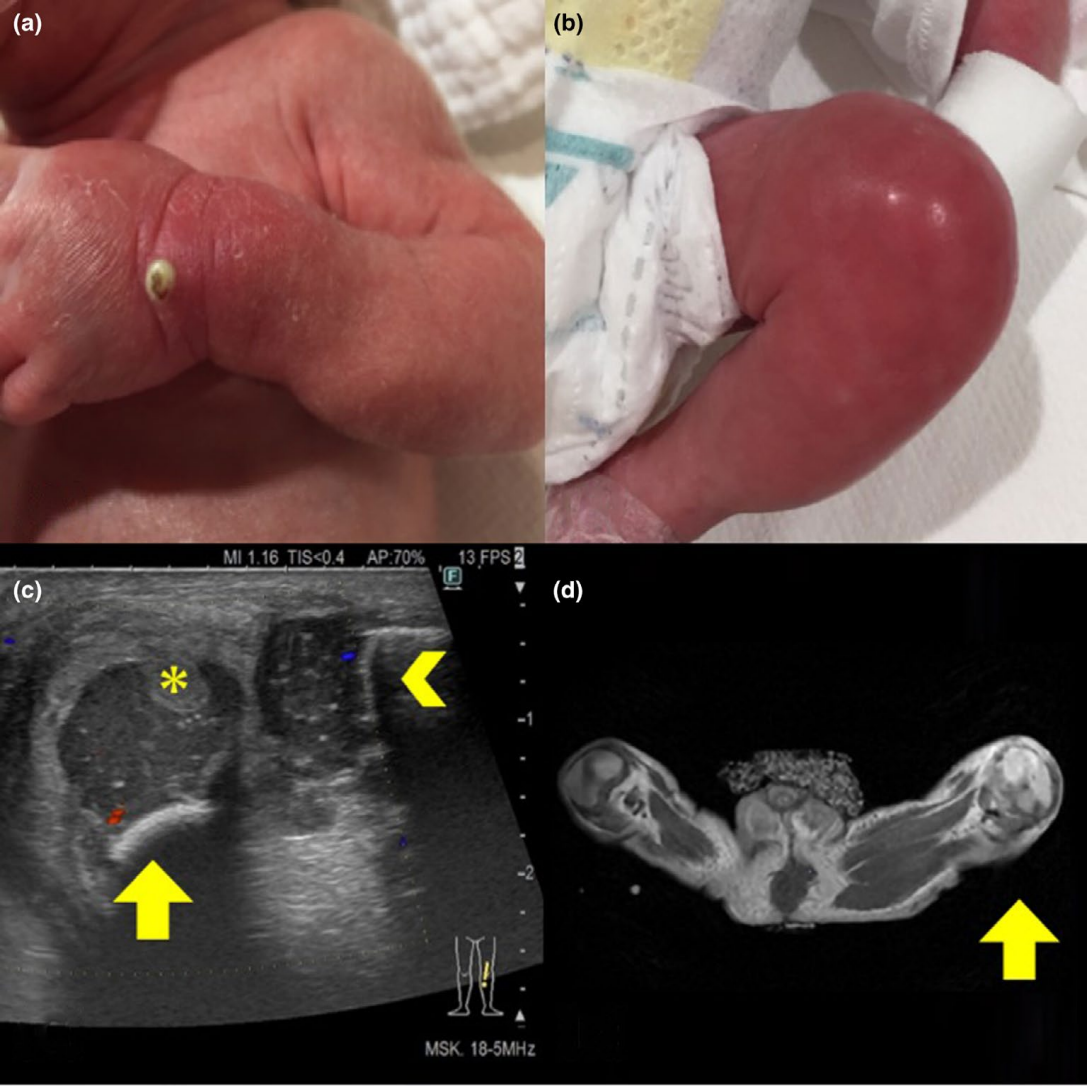
(a) On the day of life 23, redness and swelling accompanied by an accumulation of pus were found on the infant's left hand where peripheral venous catheter had been placed from the day of life 18 to 21. (b) On the day of life 28, redness and swelling appeared on the neonate's left knee. (c) Ultrasound examination performed on the day of life 43 shows femoral epiphyseal cartilage (arrow) and tibial epiphyseal cartilage (>). Increased blood flow signal is seen around the knee joint and within the epiphyseal cartilage compared to the contralateral side. Thickened synovial membrane in the anterior region partially erodes into the femoral epiphyseal cartilage (*). Inflammation from the arthritis of knee joint is extended to the distal epiphyseal cartilage implying chondritis. (d) Magnetic resonance imaging performed on the day of life 41 shows the extension of inflammation to the cartilage on the neonates's left knee (arrow) compared to the right.

On the day of life 27, the neonate showed poor general appearance. Laboratory tests revealed white blood cell count of 8300/μL (stab cell 7.5%, segmented cell 53.0%, lymphocyte 20.0%), C‐reactive protein (CRP) of 5.65 mg/dL, and platelet count of 188,000/μL. Tazobactam/piperacillin and vancomycin were started. On the day of life 28, redness and swelling appeared on the left knee (Figure 1b). Antibiotics were ineffective and ultrasound examination on the day of life 29 revealed a hyperechoic area around the knee joint. Joint aspiration detected gram‐positive cocci, confirming septic arthritis.

The neonate underwent surgical debridement and MSSA was isolated from the synovial membrane, consequently changing antibiotics to cefazolin monotherapy (150 mg/kg/day). MSSA showed the same antibiotic susceptibility as the MSSA detected from the PVC‐site, suggesting hematogenous spread from the PVC site to the knee joint. Although all blood cultures remained negative, previous studies report low sensitivity of blood cultures in neonatal septic arthritis.^5^

On postoperative day (POD) 1, CRP decreased from 11.67 mg/dL (POD 2) to 2.78 mg/dL (POD 1). Ultrasound and magnetic resonance imaging revealed inflammation extending to the cartilage (Figure 1c,d), prompting a 6‐week antibiotic course. By POD 31, spontaneous leg movement resumed. The neonate was discharged on the day of life 93 without signs of recurrence or sequelae. As of 10 months, knee joint mobility remains normal, though long‐term follow‐up is essential to detect potential motor dysfunction or deformity.

Prematurity, umbilical vessel catheter and CVC placement, femoral blood sampling, and maternal infection are known risk factors for neonatal septic arthritis.^3^ PVCs, while commonly used in NICUs, has not been recognized as a major risk. Our case illustrates that PVCs can lead to severe infections. Insufficient skin barrier function due to the neonate's prematurity, and low maternal antibodies due to the neonate's prematurity and placental dysfunction by the mother's HDP are possible contributing factors for the origination of arthritis. Born as an extremely low birthweight (ELBW) infant and a small‐for‐gestational‐age infant, he was particularly vulnerable to bacterial infections. There was no evidence of immunodeficiency.

Not only CVCs but also PVCs require special attention to prevent severe bacterial infection including septic arthritis, especially in ELBW infants due to their low immune function.

## AUTHOR CONTRIBUTIONS

A.H. wrote the initial draft and T.A. and M.F. revised and approved the final draft; M.O., K.M., and M.H. performed perinatal diagnostics and reviewed the manuscript; T.M. performed the operation. All authors reviewed the manuscript and approved the final manuscript.

## INFORMED CONSENT

Written informed consent was obtained from the patient's parents for the publication of this case report.

## CONFLICT OF INTEREST STATEMENT

The authors declare no conflict of interest.
